# The Impact of Surface Drug Distribution on the Acoustic Behavior of DOX-Loaded Microbubbles

**DOI:** 10.3390/pharmaceutics13122080

**Published:** 2021-12-04

**Authors:** Chia-Wei Lin, Ching-Hsiang Fan, Chih-Kuang Yeh

**Affiliations:** 1 Department of Biomedical Engineering and Environmental Sciences, National Tsing Hua University, Hsinchu 30013, Taiwan; s106012801@m106.nthu.edu.tw; 2 Department of Biomedical Engineering, National Cheng Kung University, Tainan 70101, Taiwan; 10908013@gs.ncku.edu.tw; 3 Medical Device Innovation Center, National Cheng Kung University, No. 1, University Road, Tainan 70101, Taiwan

**Keywords:** microbubbles, doxorubicin, stable cavitation, inertial cavitation, lipid fluidity

## Abstract

Previous studies have reported substantial improvement of microbubble (MB)-mediated drug delivery with ultrasound when drugs are loaded onto the MB shell compared with a physical mixture. However, drug loading may affect shell properties that determine the acoustic responsiveness of MBs, producing unpredictable outcomes. The aim of this study is to reveal how the surface loaded drug (doxorubicin, DOX) affects the acoustic properties of MBs. A suitable formulation of MBs for DOX loading was first identified by regulating the proportion of two lipid materials (1,2-distearoyl-sn-glycero-3-phosphocholine (DSPC) and 1,2-distearoyl-sn-glycero-3-phospho-rac-glycerol sodium salt (DSPG)) with distinct electrostatic properties. We found that the DOX loading capacity of MBs was determined by the proportion of DSPG, since there was an electrostatic interaction with DOX. The DOX payload reduced the lipid fluidity of MBs, although this effect was dependent on the spatial uniformity of DOX on the MB shell surface. Loading DOX onto MBs enhanced acoustic stability 1.5-fold, decreased the resonance frequency from 12–14 MHz to 5–7 MHz, and reduced stable cavitation dose by 1.5-fold, but did not affect the stable cavitation threshold (300 kPa). Our study demonstrated that the DOX reduces lipid fluidity and decreases the elasticity of the MB shell, thereby influencing the acoustic properties of MBs.

## 1. Introduction

Microbubbles (MBs) with lipid shells and gaseous contents have been proposed as attractive drug carriers for achieving noninvasive diagnosis, therapy, and localized drug delivery in biological tissues, in combination with ultrasound (US). However, translation of these findings to the clinic has been hindered by the naturally low drug capacity of MBs and the unknown acoustic properties of MBs after drug loading. The use of MBs mainly relies on the dynamic behavior of MBs in response to US waves, in particular their expansion, contraction, and collapse, a process termed cavitation. In response to low-intensity US, MBs undergo a steady oscillation (stable cavitation) that can induce reversible vascular permeabilization with a low level of cargo release. High-intensity US triggers large-amplitude and asymmetric oscillation of MBs, and ultimately violent collapse (inertial cavitation). The inertial cavitation of MBs can totally release their cargo and concurrently produces a strong mechanical stress that damages nearby tissues. In addition, the MBs also emit nonlinear harmonic signals when stimulated at their resonance frequency with low-intensity US, providing a unique opportunity to distinguish between MBs and tissue echoes for background-free US molecular imaging. Exploiting such acoustic properties for drug-loaded MBs with a high payload could allow the precise control of the MBs to generate desired behaviors and expand medical applications.

In this study, we focused on loading doxorubicin (DOX), a clinically used chemotherapy drug that is usually associated with high cardiotoxicity [1], onto the MB shell. The side effects of DOX could potentially be largely reduced, and its treatment efficiency could be improved by integrating DOX into MBs with subsequent US excitation. To fabricate DOX-MBs, DOX could be pre-prepared as a liposome formulation [2] and then tethered to the outer shell of MBs, or synthesized in a hydrophobic form for incorporation into MB shells [3]. The major concerns of this approach are a potential immune response, prolonged circulation time of liposomal DOX (0.4 h to 60 h) [2,4], and the unknown pharmacokinetics of the modified drug. To address these issues, we aimed to develop a unique MB formulation that would allow direct loading of DOX onto MBs via regulation of the lipid components of the MB shell. Given the cationic property of DOX [3], two lipid materials with distinct electrostatic properties, 1,2-distearoyl-sn-glycero-3-phosphocholine (DSPC) and 1,2-distearoyl-sn-glycero-3-phospho-rac-glycerol sodium salt (DSPG), were selected for preparation of MBs to investigate their DOX affinity. Although several formulations of DOX-MBs have been proposed [3,5,6], there is little information on the relationship between the DOX payload and the surface charge of MBs. In addition, it is essential to characterize the properties (e.g., size distribution, concentration and DOX payload) of the DOX-MBs before use.

Previous studies have observed that the acoustic properties of MBs are changed upon integration of cargos. Chang et al. observed that the dissolution of MBs was accelerated after incorporation of anti-VEGFR2 antibodies onto the MB shells [7]. Gu et al. noticed that both the stable and inertial cavitation thresholds of superparamagnetic iron oxide nanoparticle (SPIO)-loaded MBs decreased with increasing SPIO concentration [8]. One recent study showed that as more SPIOs were integrated onto the MB shell, the mean radius of SPIOs-MBs increased and the stiffness of the MB shell decreased, which in turn resulted in an enhanced cavitation intensity and a weakened resistance to MB collapse [9]. However, to date, no study has reported the effect of a chemotherapeutic drug (e.g., DOX, 580 Da molecular weight) on the acoustic behaviors of MBs.

The stiffness of the MB shell has been reported as another key factor determining the acoustic characteristics of MBs [10,11,12]. The concept of shell stiffness covers several dynamic properties of a phospholipid layer, including chain flexing, molecular wobbling, and lateral diffusion of molecules [13,14]. Decreased stiffness of the MB shell may result in a less-ordered membrane structure, enhancing MB dissolution and collapse [15]. Therefore, we speculated that the integration of DOX into the MB shell would probably change the stiffness of the shell and thus affect the acoustic behaviors of the MBs. To test this hypothesis, we first altered the proportion of lipid components to adjust the surface charge of MBs and maximize the DOX payload of MBs with a high MB yield. The effect of DOX on the acoustic properties of MBs and the potential underlying mechanisms were then investigated. We assessed acoustic stability by B-mode imaging, cavitation response by passive cavitation detection, and resonance frequency by acoustic attenuation. This work provides a better understanding of how the integration of DOX into the shells affects MBs, for the further development of precise clinical applications.

## 2. Materials and Methods

This study was divided two sections: (1) fine-tuning the proportion of MB lipid components to maximize DOX payload of MBs with a high MB yield, and (2) investigation of whether the acoustic properties of MBs are affected by the loaded DOX.

### 2.1. Experiment A: Fine-Tuning the Proportion of MB Lipid Components to Maximize DOX Payload of MBs with a High MB Yield

#### Preparation of DOX-Loaded MBs

All lipids (DSPC, DSPG and 1,2-distearoyl-sn-glycero-3-phosphoethanolamine-*N*-[methoxy(poly(ethylene glycol))-2000] (DSPE-PEG2000)) were purchased from Avanti Polar Lipids (Avanti Polar Lipids, Birmingham, AL, USA). DSPC, a long acyl chain lipid, enhanced the stability of the MB formulations in the human circulation [16,17]. DSPE-PEG2000 was used to maintain MB dispersibility and biocompatibility in a physiological environment [18]. DSPG, a positively charged lipid, was expected to have a high affinity for DOX by electrostatic interaction [3]; therefore, increasing the incorporation of DSPG within the lipid membrane of MBs would potentially improve the DOX payload of MBs. We adjusted the DSPC:DSPG:DSPE-PEG2000 mass ratios in six MB formulations (designated F1–F6, Table 1) to vary the positive charge for optimal loading of DOX.

**Figure 1:**
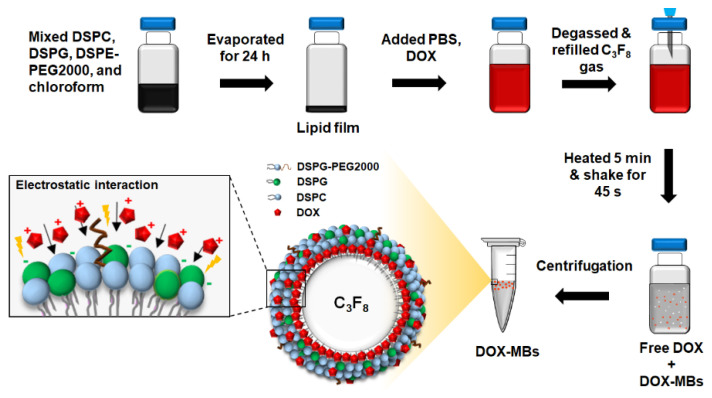
Flow chart of preparation of DOX-MBs and schematic showing the structure of DOX-MBs. DSPC and DSPE-PEG2000 were neutral charged lipids. DSPG was a positively charged lipid, which was expected to have a high affinity for anionic DOX by electrostatic interaction. We hypothesized that increasing the incorporation of DSPG within the lipid membrane of MBs would potentially improve the DOX payload of MBs. We adjusted the DSPC:DSPG:DSPE-PEG2000 mass ratios in six MB formulations for optimal loading of DOX.

Each material was dissolved homogeneously in chloroform and eliminated using an evaporator (R-210, Büchi Labortechnik AG, Flawil, Switzerland) to form a thin film (Figure 1). A 0.5 wt% glycerol containing phosphate-buffered saline (PBS) was mixed with the film and dispersed with a sonicator (Model 2510, Branson Ultrasonics Corp., Danbury, CT, USA) at 60 °C. A total of 1500 μg DOX was mixed into the lipid solution for 1 h at 60 °C because MBs hardly formed when more than 1500 μg DOX was added (data not shown). Subsequently, the mixture was degassed and refilled with perfluoropropane (C3F8). Finally, DOX-MBs were formed by agitation at 4550 rpm for 45 s. To remove the unreacted DOX and lipids, the DOX-MBs were centrifuged at 500× *g* for 2 min and washed four times with 0.5 wt% glycerol-PBS. Comparable MBs without addition of DOX were prepared for comparison.

DSPC, a long acyl chain lipid, enhanced the stability of the MB formulations in the human circulation. DSPE-PEG2000 was used to maintain MB dispersibility and biocompatibility in a physiological environment. DSPG, a positively charged lipid, was expected to have a high affinity for DOX by electrostatic interaction; therefore, increasing the incorporation of DSPG within the lipid membrane of MBs would potentially improve the DOX payload of MBs. We adjusted the DSPC:DSPG:DSPE-PEG2000 mass ratios in six MB formulations (designated F1–F6, Table 1) to vary the positive charge for optimal loading of DOX.

### 2.2. Properties of the DOX-MBs

#### Size Distribution, Concentration and Payload

The structure of DOX-MBs is illustrated in Figure 1. The size distribution as well as concentration of the DOX-MBs were estimated by a Coulter counter (Multisizer 3, Beckman Coulter, Brea, CA, USA). The surface charge of MBs with and without DOX loading was determined by Zetasizer (model Nano ZS, Malvern Instruments, Worcestershire, UK). The DOX distribution on MBs was observed using a confocal fluorescence microscope (LSM 800, Zeiss, Oberkochen, Germany) with a 60× oil objective (Zeiss). To measure the DOX payload of DOX-MBs, the DOX-MBs were disrupted using a sonicator (2510, Branson Ultrasonics, Danbury, CT, USA) and the absorbance of DOX at 490 nm was measured with a spectrophotometer (Infinite^®^ 200PRO series, Tecan, Männedorf, Switzerland). The loading efficiency of DOX on MBs was estimated based on the amount of DOX loaded on DOX-MBs as a percentage of the total amount of added DOX. Note that the absorbance values of DOX-MBs solutions were converted to concentration of DOX using a standard linear calibration curve.

The DOX intensity projection on an x-y image plane by confocal fluorescence microscope was used to quantify the DOX distribution (uniformity) onto the shell of DOX-MBs. The image was divided into 64 sub-images (square size of 10 pixels by 10 pixels) (Figure S1). Then, the mean DOX signal (red fluorescence) in each sub-image was calculated by MATLAB^TM^ software (Version 9.0.0.341360 (R2016a), The MathWorks, Natick, MA, USA). The deviation of each MB referred to the uniformity of the DOX distribution onto the shell of DOX-MBs.

### 2.3. Experiment B: Investigate the Impact of DOX Payload on the Acoustic Properties of MBs

#### 2.3.1. Membrane Fluidity

To measure the membrane fluidity of MBs, DOX-MBs labeled with a spin-labeled probe were prepared. Briefly, 2.2 mg of 1-palmitoyl-2-stearoyl-(5-doxyl)-sn-glycero-3-phosphocholine (Avanti Polar Lipids) was added to the lipid mixture (0.25% of total lipid mole) in chloroform prior to the generation of DOX-MBs, as described above.

The membrane fluidity of MBs after loading different doses of DOX (0–3000 μg) was determined by electron paramagnetic resonance (EPR) spectroscopy (Bruker ELEXSYS E580 −400 CW/Pulsed spectrometer, Bruker, Rheinstetten, Germany) with a helium gas flow system (4118CF and 4112HV) and a split-ring resonator (EN4118X-MS3, Bruker). The operated parameters were as follows: modulation amplitude, 1.6 G; modulation frequency, 100 kHz; microwave power, 15 mW; magnetic field scan, 199.9 G; sampling time, 20.3 ms; and receiver time constant, 32.7 ms.

#### 2.3.2. Acoustic Stability

**Figure 2:**
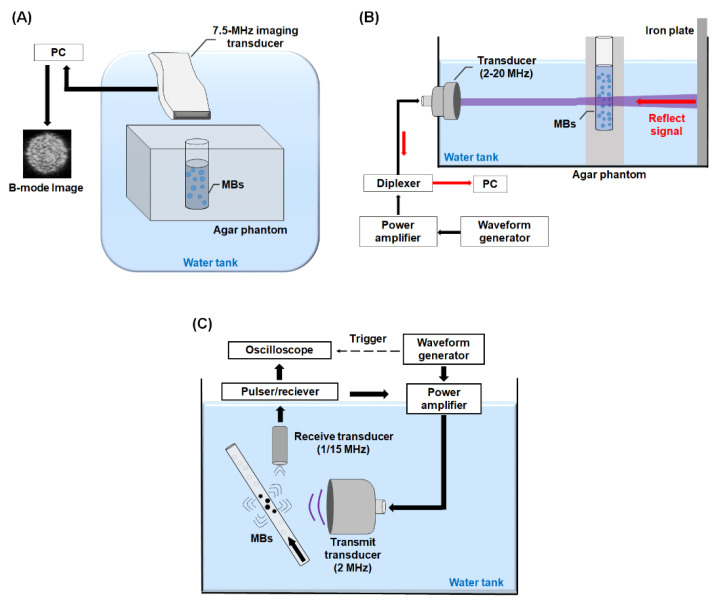
Experimental setup for measuring (**A**) acoustic stability, (**B**) resonance frequency, and (**C**) cavitation activities of DOX-MBs.

The echogenicity of the DOX-MBs from ultrasonic B-mode images was used to estimate the acoustic stability of the DOX-MBs. DOX-MBs (2.5 × 10^7^ MBs/mL) were loaded into a chamber within a 2% agarose phantom and imaged via a 7.5 MHz ultrasonic imaging system (model t3000, Terason, Burlington, MA, USA) (Figure 2A). The B-mode images were obtained at an interval of 10 min for 1 h at 37 °C. For comparison, the acoustic stability of pure-lipid MBs and DOX-MBs was also monitored by ultrasound contrast enhancement imaging with a commercial ultrasound imaging system (Ultrasoundscript 3, Ecare, Shanghai, China). The contrast-to-noise ratios (CNRs) of MBs from each image were analyzed via MATLAB^TM^ software as the echogenicity of MBs. The CNR was denoted as the backscatter signal of samples divided by background signal (agarose phantom). The CNR value at each time point was normalized to that at 0 min for comparison.

#### 2.3.3. Resonance Frequency

When US waves propagate through a MB suspension, the US energy is scattered and absorbed, resulting in the attenuation of a reflected signal from a reference reflector. It was shown that the acoustic attenuation of MBs is a function of frequency and the maximal attenuation regimen could be denoted by the resonance frequency of MBs. Therefore, the resonance frequency of the DOX-MBs generated in this study was estimated by the pulse-echo substitution method [19]. The transducers were triggered by a waveform generator (AWG 2040, Tektronix, Beaverton, OR, USA) and an RF power amplifier (325LA, E&I, Rochester, NY, USA) to excite DOX-MBs with a cycle number of 30, repetition frequency of 100 Hz, and acoustic pressure of 200 kPa. The experimental setup is illustrated in Figure 2B. Briefly, several ultrasound transducers (V307-V380, Olympus panametrics-NDT, Tokyo, Japan) were utilized to cover the range of 2–20 MHz (Table 2). The DOX-MBs were loaded into an ultrasound-penetrable agarose chamber (wt%: 2%) located within the US beam of each transducer, which entered the chamber and was then reflected off a steel plate located at the focus. The reflected signal was received by the transmitted ultrasound transducers and sent to the PC through a diplexer (RDX-6, RITEC Inc., Warwick, RI, USA). Finally, the signal was processed by MATLAB^TM^ software. To avoid the scattering effect of MBs, the DOX-MBs were diluted to a concentration of 2.0 × 10^5^ MBs/mL [20,21]. The attenuation coefficient of DOX-MBs at each frequency was quantitated as follows:

$$\mathrm{Attenuation}\;\mathrm{coefficient}\left(\mathrm{dB}/\mathrm{mm}\right)=-\frac{20}{L}\log_{\;}\left(\frac{P_{\mathrm{MBs}}}{P_{\mathrm{PBS}}}\right)$$

where L is the distance of phantom chamber, P_MBs_ is peak negative pressure of the signal attenuated by DOX-MBs and P_PBS_ is the peak negative pressure of the signal attenuated by PBS.

Prior to starting the experiments, a polyvinylidene difluoride type hydrophone (model HGL-0085, ONDA, Sunnyvale, CA, USA) was used to measure the acoustic pressures of each transducer in a water tank filled with degassed and distilled water at 25 °C.

#### 2.3.4. Cavitation Activities

##### Stable Cavitation

Previous studies reported that the cavitation activities of MBs could be quantified from the acoustic-emission signals when the MBs were sonicated by US [22,23]. Therefore, a passive cavitation method was used to collect the acoustic-emission signals emitted by MBs. The experimental setup is illustrated in Figure 2C. For measurement of stable cavitation activities, the DOX-MBs (5 × 10^8^ MBs/mL) were injected into a cellulose tube (diameter: 200 µm). The injection velocity was set at 10 mL/h by a syringe pump (KDS120, KD Scientific, New Hope, PA, USA). The DOX-MBs were then sonicated by a 2 MHz focused US transducer (SU-101, Sonic concepts, Bothell, WA, USA) with a cycle number of 500, pulse repetition frequency of 100 Hz, and acoustic pressure of 0–1000 kPa, whereas the acoustic-emission signal of MBs was acquired via a 1 MHz focused US transducer (V303, Olympus panametrics-NDT). The signals were then magnified through a pulser/receiver (5072PR, Olympus panametrics-NDT) and acquired via an oscilloscope (LT322, Teledyne Technologies, Thousand Oaks, CA, USA). The 2 MHz focused US transducer was triggered by the abovementioned waveform generator and the RF power amplifier.

**Figure 3:**
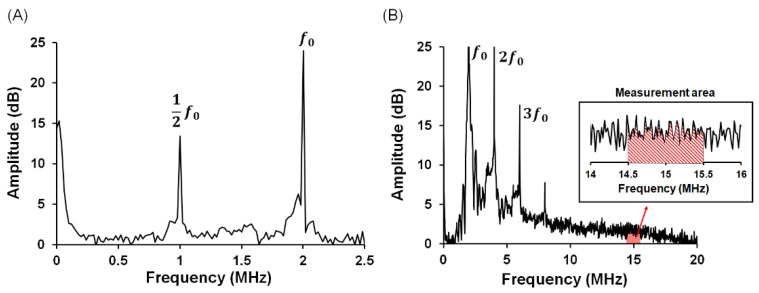
The frequency spectra for calculating (**A**) stable cavitation dose and (**B**) inertial cavitation dose of cavitating DOX-MBs.

The acquired signals were subjected to fast Fourier transformation using MATLAB^TM^ software. The subharmonic frequency component (1/2F0, 1.0 MHz) within the spectra indicated the occurrence of stable cavitation (Figure 3A). The stable cavitation dose was quantified by the peak intensity difference between the fundamental signal (F0) and subharmonic signal [24].

##### Inertial Cavitation

To measure inertial cavitation activities, the receiving transducer was replaced with a 15 MHz focused US transducer (V319, Olympus). The wideband signal within spectra indicated the occurrence of inertial cavitation. The inertial cavitation dose was calculated as the area within the bandwidth of the received transducer (about 14.5–15.5 MHz) [23], but excluded the transducer’s harmonic and ultraharmonic frequencies (Figure 3B).

### 2.4. Statistics

All data expressed as means and standard deviations were measured from at least three independent experiments. Statistical analysis was performed with two-tailed Student’s t-test or one-way ANOVA. A *p*-value less than 0.05 (*p* < 0.05) was considered a significant difference. Correlations were evaluated using Pearson’s correlation coefficients. Calculations were performed with the SPSS software package (Version SPSS statistics 25, SPSS Inc., Chicago, IL, USA).

## 3. Results

### 3.1. Characterization of DOX-MBs

**Figure 4:**
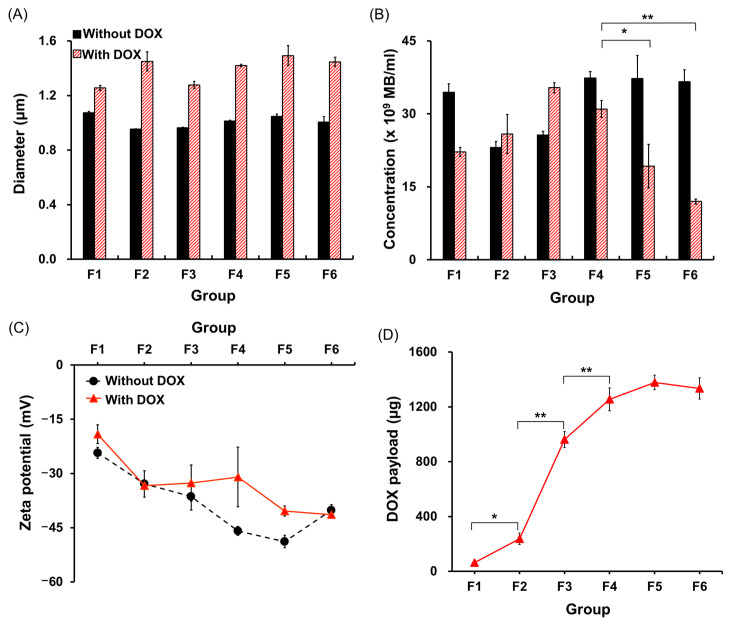
(**A**) Diameter, (**B**) concentration, and (**C**) zeta-potential for each MB formulation with and without DOX loading. (**D**) DOX payload of each MB formulation. (*, *p* < 0.05; **, *p* < 0.005; *n* = 3 for each test).

An ideal DOX-MB for theranostic application must meet the following criteria: small size (smaller than 6 µm, to avoid gas embolism in capillaries [25]), high MB yield (in vivo safe administration dose: 4 × 10^10^ MBs/rat [26]) and high DOX payload. We therefore first characterized the properties of different lipid formulations, including mean size, concentration, DOX payload, and surface charge. Figure 4A shows that mixing DSPG into the MB lipid shell did not affect MB size (F1: 1.07 ± 0.01 μm; F2: 0.95 ± 0.00 μm; F3: 0.96 ± 0.01 μm; F4: 1.01 ± 0.01 μm; F5: 1.05 ± 0.02 μm; F6: 1.01 ± 0.04 μm). Although larger MBs were formed when DOX (1500 μg) was added into MBs, there was no statistical difference between the formulations (F1: 1.26 ± 0.02 μm; F2: 1.45 ± 0.07 μm; F3: 1.28 ± 0.03 μm; F4: 1.42 ± 0.01 μm; F5: 1.49 ± 0.07 μm; F6: 1.45 ± 0.03 μm). Compared with the non-DSPG group (F1), formulations with a small amount of DSPG produced a lower concentration of MBs (F1: 34.4 ± 1.7 × 10^9^ MBs/mL; F2: 23.1 ± 1.2 × 10^9^ MBs/mL; F3: 25.6 ± 0.7 × 10^9^ MBs/mL) (Figure 4B). A further increase in the amount of DSPG improved the MB concentration to levels similar to that of F1 (F4: 37.3 ± 1.3 × 10^9^ MBs/mL; F5: 37.2 ± 4.7 × 10^9^ MBs/mL; F6: 36.6 ± 2.5 × 10^9^ MBs/mL). However, the presence of DOX reduced the yield of MBs for both low and high proportions of DSPG (F1: 22.1 ± 0.9 × 10^9^ MBs/mL; F2: 25.8 ± 4.0 × 10^9^ MBs/mL; F5: 31.0 ± 1.7 × 10^9^ MBs/mL; F6: 11.9 ± 0.5 × 10^9^ MBs/mL), and only F3 and F4 could produce high concentrations of MBs (F4: 31.0 ± 1.7 × 10^9^ MBs/mL; F3: 35.4 ± 1.0 × 10^9^ MBs/mL).

As expected, the surface charge of MBs decreased as the proportion of DSPG increased (F1: −24.3 ± 1.4 mV; F2: −32.9 ± 3.6 mV; F3: −36.4 ± 3.7 mV; F4: −45.9 ± 1.2 mV; F5: −48.8 ± 1.7 mV; F6: −40.2 ± 1.5 mV), due to the negatively charged hydroxyl group of DSPG. The higher surface charge of F6 compared with F5 is probably due to the excessive DSPG lipid causing MB aggregation, which interfered with the measurement. After the addition of DOX, an increase in MB surface charge was observed for all formulations (F1: −19.7 ± 2.5 mV; F2: −33.4 ± 0.8 mV; F3: −32.6 ± 5.0 mV; F4: −30.9 ± 8.2 mV; F5: −40.3 ± 1.4 mV; F6: −41.4 ± 0.3 mV), confirming the successful loading of DOX onto the MB shell (Figure 4C).

The DOX loading capability of MBs was also affected by the proportion of DSPG: the DOX payload increased as the amount of DSPG increased (F1: 63.1 ± 7.1 μg; F2: 236.9 ± 41.4 μg; F3: 962.2 ± 58.1 μg; F4: 1254.7 ± 83.5 μg). When more DSPG was incorporated into the MBs, the DOX payload plateaued at 1377.7 ± 52.3 μg and 1333.6 ± 77.3 μg in F5 and F6, respectively, consistent with the observations of surface charge (Figure 4D). Taken together, these findings identified F4 as a suitable formulation of DOX-MBs for use in subsequent experiments because of its high DOX loading capability with a high MB yield.

### 3.2. DOX Distribution and Membrane Fluidity of DOX-MBs

**Figure 5:**
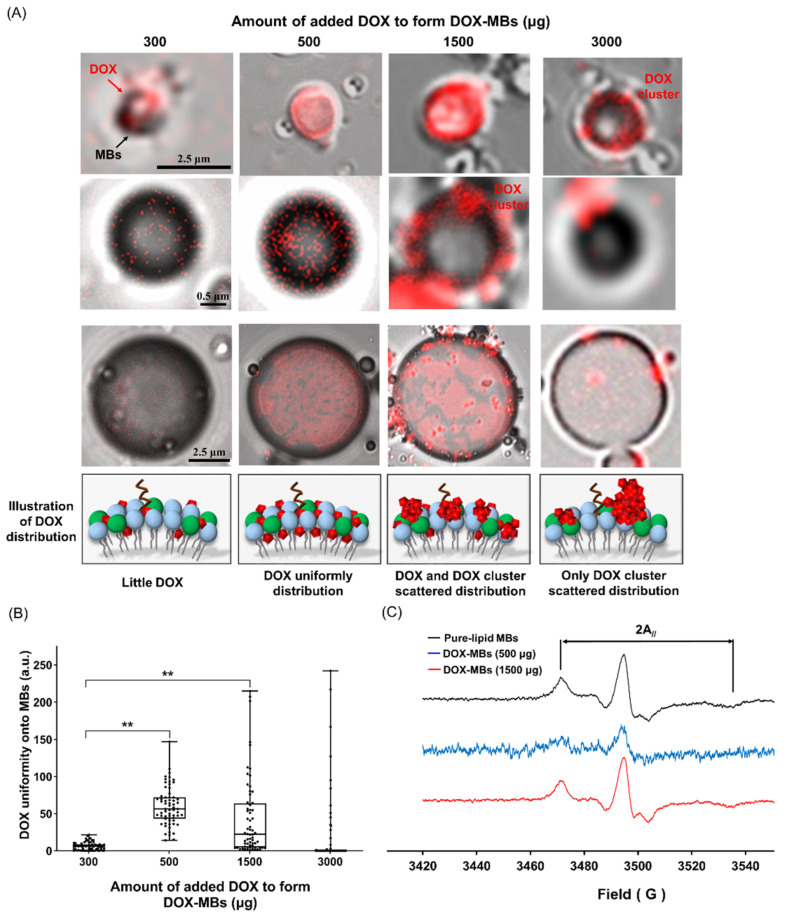
(**A**) Confocal microscopic images of DOX-MBs with DOX payload from 0 to 3000 μg; *n* = 5. (**B**) The quantitative uniformity of DOX on MBs shell; *n* = 5. (**C**) The lipid fluidity of DOX-MBs with DOX payload of 0, 500 μg and 1500 μg measured by EPR; *n* = 1. (**, *p* < 0.005).

After characterizing the suitable MB formulation for DOX loading, we next investigated whether the acoustic properties of MBs were affected by the DOX payload. Serial doses of DOX (0–3000 μg) were used to synthesize DOX-MBs and pure-lipid MBs without DOX loading were included as a comparison. The DOX payload of these doses was 107.3 ± 88.3 μg (300 μg group), 184.4 ± 39.3 μg (500 μg group), 1254.7 ± 39.3 (1500 μg group) and 1024.5 ± 706.2 μg (3000 μg group), respectively. Because the shell property of MBs is highly correlated with MB acoustic properties, we used microscopic imaging and EPR spectroscopy to observe the morphology and membrane fluidity of the DOX-MBs. Figure 5A shows the morphology of the DOX-MBs by fluorescence and bright-field microscopic imaging. Co-localization of MB morphology and DOX fluorescence signals indicated successful loading of DOX onto the shell of MBs. A uniform but weak DOX signal was visualized on the shell of MBs in the 300 μg group, since the low dose of DOX (5.9 ± 5.3 a.u, Figure 5B). The layer of DOX fluorescence in the 1500 μg group was denser and thicker than that in the 500 μg group, consistent with the quantitative data. However, the lower deviation of DOX fluorescence signals in the 500 μg group than the 1500 μg group (60.1 ± 25.5 a.u. vs. 43.4 ± 51.7 a.u.) suggested that the DOX distribution on MBs was more uniform in the 500 μg group. We further observed several scattered DOX clusters located on the outer surface of the MB shell when the dose of DOX was increased to 3000 μg, which might be attributed to self-aggregation of DOX before attaching to the MBs at such a high concentration. In the meantime, the uniformity of DOX on the MBs shell was also largely reduced (19.2 ± 49.1 a.u). Because of the extremely low DOX payload and low DOX uniformity observed in the 300 μg group and 3000 μg group, we selected only the 0 μg (pure-lipid MBs), 500 μg and 1500 μg groups for the following experiments.

The EPR analysis suggested that the membrane fluidity of pure-lipid MBs was 32.1 G. The membrane fluidity of the 1500 μg group (32.1 G) was close to that of pure-lipid MBs, but higher than that in the 500 μg group (25.7 G) (Figure 5C). Previous studies observed from electronic spin resonance spectroscopy had reported that the intensity of 2A//peak was associated with a decrease in fluidity [27]. Based on these two distinct observations, it appeared that the membrane fluidity of MBs was dependent on the distribution of DOX on its shell, and the membrane fluidity of MBs was largely restricted when DOX was incorporated evenly into the lipid bilayer of the MB shell.

### 3.3. Acoustic Properties of DOX-MBs

**Figure 6:**
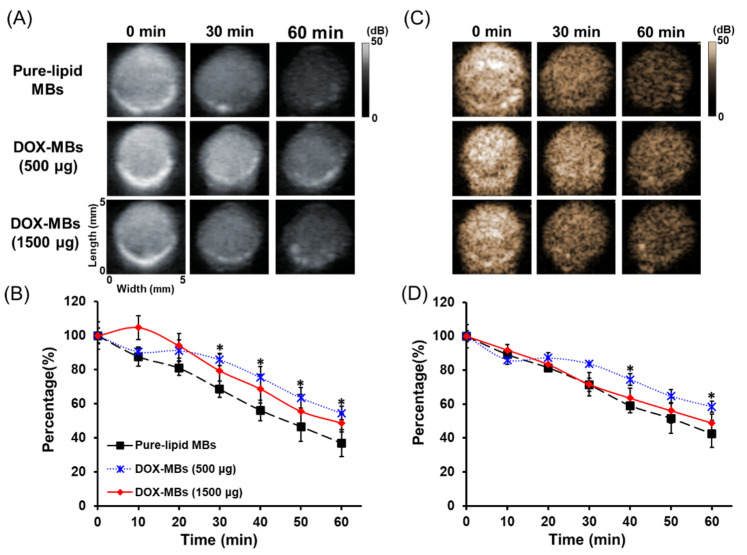
Acoustic stability of pure-lipid MBs and DOX-MBs. (**A**) Ultrasound B-mode images of pure-lipid MBs and DOX-MBs at different time points. (**B**) The corresponding quantitative data. (**C**) Ultrasound contrast enhancement images of pure-lipid MBs and DOX-MBs at different time points. (**D**) The corresponding quantitative data (*, *p* < 0.05; *n* = 3 for each test).

Given that the DOX distribution and membrane fluidity of MBs varied according to the dose of DOX, we first asked whether the acoustic stability was influenced by these two factors. The presence of a gaseous core inside MBs plays an important role in US-mediated MB drug release. Because the contrast enhancement of B-mode imaging of MBs is mainly controlled by the gaseous core inside, the sonographic imaging was employed to assess the stability of DOX-MBs with different DOX payload. The number percentage of pure-lipid MBs gradually declined with time (0 min: 100.0 ± 4.0%; 50 min: 47.1 ± 3.7%) (Figure 6A), suggesting the natural gas diffusion from MBs. The DOX-MBs showed statistically higher acoustic stability than pure-lipid MBs; however, there was no significant difference between the two DOX groups at each time point (0 min: 100.0 ± 1.6% for 500 μg, and 100.0 ± 2.5% for 1500 μg; 60 min: 63.5 ± 9.1% for 500 μg and 72.6 ± 4.6% for 1500 μg) (Figure 6B). The contrast enhancement images showed that the signal intensity (%) of pure-lipid MBs declined with time (0 min: 100.0 ± 1.6%; 60 min: 42.4 ± 7.9%), which was similar with the DOX-MBs (1500 μg) group (0 min: 100.0 ± 3.3%; 60 min: 48.8 ± 5.2%) (Figure 6D). The DOX-MBs (500 μg) showed a consistent trend with those two groups at 0 min, 10 min, and 20 min, but a statistically higher signal intensity than those two groups at the following time point (74.5 ± 3.3% and 58.3 ± 3.2% for 40 min and 60 min) (Figure 6D). These data suggested that the loading of DOX probably reduced the natural gas diffusion from MBs, but increasing the dose of DOX did not further prolong the acoustic stability of MBs. The gas diffusion of MBs was not affected by the DOX distribution and the membrane fluidity of MBs.

**Figure 7:**
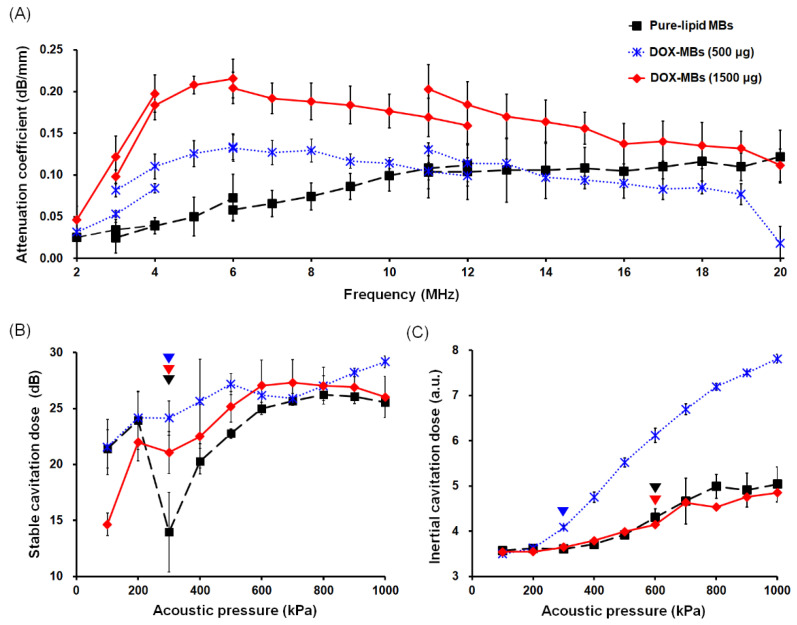
(**A**) Resonance frequency of pure-lipid MBs and DOX-MBs. (**B**) Stable cavitation dose of pure-lipid MBs and DOX-MBs with different acoustic pressure (0–1000 kPa) of US sonication. (**C**) Inertial cavitation dose of pure-lipid MBs and DOX-MBs with different acoustic pressure (0–1000 kPa) of US sonication. (*n* = 3 for each test).

Figure 7A demonstrates the resonance frequency of pure-lipid MBs and DOX-MBs. For pure-lipid MBs, high attenuation coefficients were obtained in the frequency range of 12–14 MHz, showing that pure-lipid MBs had a peak resonance frequency at this region. For DOX-MBs, the major attenuation appeared at 5–7 MHz. However, there was no significant difference between the two doses, and further increasing the dose of DOX did not further reduce the resonance frequency of MBs. Because the resonance frequency of MBs was dominated more by their size than by their shell properties, it is reasonable that the resonance frequency of pure-lipid MBs (mean size: 1.1 ± 0.1 μm) was higher than that of DOX-MBs (mean size: 1.3 ± 0.1 μm for 500 μg group and 1.4 ± 0.1 μm for 1500 μg group).

We then investigated the cavitation activities of pure-lipid MBs as well as DOX-MBs. The acoustic pressure threshold for stable cavitation and inertial cavitation of pure-lipid MBs was 300 kPa and 600 kPa, respectively (Figure 7B,C). The stable cavitation dose of pure-lipid MBs was 13.9 ± 3.5 dB. Because the stable cavitation dose was quantified by the difference in peak intensity between the fundamental signal (F0) and subharmonic signal (1/2F0), such a low value represents the high oscillation property of pure-lipid MBs. The threshold for stable cavitation of DOX-MBs was also 300 kPa. However, a significantly higher stable cavitation dose (24.1 ± 1.5 dB) was measured during the experiment with DOX-MBs (500 μg), probably because the reduced membrane fluidity limited the vibration range of MBs. In contrast, the subharmonic frequency dose of DOX-MBs (1500 μg) was slightly lower than that of DOX-MBs (500 μg), most likely because the non-uniform distribution of DOX still provided some space for MBs’ oscillation (21.0 ± 1.8 dB).

DOX-MBs (500 μg) had an inertial cavitation threshold of 300 kPa and the inertial cavitation doses were obviously higher than those of the other groups (4.08 ± 0.03 a.u. for 500 μg; 3.64 ± 0.04 a.u. for 1500 μg; 3.60 ± 0.01 a.u. for pure-lipid MBs) (Figure 7C). This is probably because the reduced lipid fluidity due to DOX would decrease the elasticity of the MB shell, making the MBs easier to break. In contrast, DOX-MBs (1500 μg) exhibited a similar acoustic threshold of inertial cavitation (600 kPa) and inertial cavitation dose to those of pure-lipid MBs (4.14 ± 0.07 a.u. for 1500 μg; 4.30 ± 0.19 a.u. for pure-lipid MBs), most likely because the MBs with a non-uniform distribution of DOX could still oscillate.

## 4. Discussion

In this study, we successfully optimized the MB formulation for high DOX payload with high MB yield. We found that the DOX payload capability of MBs was determined by the proportion of DSPG. The DOX surface distribution, membrane fluidity, and cavitation activities of MBs were affected by the DOX payload. In addition, the reduction in membrane fluidity was dependent on a uniform distribution of DOX on the MB shell. We found that loading DOX onto MBs enhanced acoustic stability 1.5-fold and decreased the resonance frequency from 12–14 MHz to 5–7 MHz, independent of the payload of DOX. Interestingly, the DOX-induced reduction in membrane fluidity increased the stable cavitation dose 1.5-fold and reduced the inertial cavitation threshold (400 kPa for DOX-MBs; 600 kPa for pure-lipid MBs). Our study demonstrates that DOX loading has only a minor influence on the acoustic properties of MBs through reduced membrane fluidity.

In clinical applications, DOX is often employed in a liposomal formulation, which can decrease cardiotoxicity and other side effects. However, the drug delivery efficiency of liposomal DOX was lower than free DOX because of the large size (~100 nm) after transferring to liposomal form. Previous studies have shown that US-induced MB cavitation can enable the delivery of DOX, either alone or encapsulated in a liposome, to tumors for a short time course. However, two injections were required for this approach, one for MBs and one for liposome-DOX and the long circulation time (~65 h) of undelivered liposome-DOX might elicit off-target effects. To address these problems, we propose an MB formulation that can directly load free DOX without further modification of the drug. Previous studies have reported that drug-loaded MBs can release their cargos via stable cavitation and inertial cavitation. Since we have characterized the acoustic properties of DOX-MBs, the anti-tumor application would be the next step of this project.

When considering clinical translation, it will be critical to deliver an effective drug dose at the tumor margin. Although the DOX payload of our proposed DOX-MBs is 3.7 times higher than that used in previous studies, it is still 12 times lower than the effective dose used in clinical application (32.4 mg for a 60 kg human) [28]. Fortunately, this shortcoming could be overcome by several existing strategies: (1) a modified targeting ligand on the outer surface of the MB shell could increase the local drug concentration from 8- to 18-fold [29,30]; (2) assisted targeting of MBs using ultrasound-induced radiation force (6- to 60-fold increase) [31,32,33,34,35,36] or cavitation (7-fold increase) [37]; (3) combined targeting of MBs with ultrasound radiation force and cavitation (3 to 27 fold increase) [38,39,40,41]. Using these methods, it might be feasible to apply our proposed DOX-MBs for anti-tumor applications. Another potential limitation of using MBs as a drug carrier is high drug leakage. We evaluated the drug retention of F4, F5 and F6 DOX-MBs at 37 °C by measuring their drug leakage over time. Only approximately a 10% DOX leakage was observed within 15–60 min in these three groups, with a dramatic increase over 120 min (F4: 16%; F5: 15%; F6: 8%) (Figure S2). Based on these findings, administering the proposed DOX-MBs into the blood circulation for in vivo application is feasible.

Our findings revealed that the acoustic properties of MBs depend on the DOX payload. Both the stable cavitation intensity and inertial cavitation dose of DOX-MBs (500 μg) were lower than those of pure-lipid MBs. A previous study observed that adding drugs (tacrolimus, clobetasol) into lipid carriers reduced the lipid fluidity of a lipid matrix [42]. Considering these findings in combination with our microscopic observations, we concluded that a uniform distribution of DOX within the lipid shell would increase the shell stiffness of MBs by reducing lipid fluidity, thereby reducing the oscillation of MBs. However, further increasing the dose of DOX to 1500 μg increased the cavitation activities of DOX-MBs to levels similar to those of pure-lipid MBs, possibly because the uneven distribution of DOX on the MB shell only slightly reduced the lipid fluidity and did not affect the oscillation ability of MBs.

## 5. Conclusions

This study demonstrated that the MB payload capacity could be controlled by regulating the anion lipid component ratio of lipid mixtures. We also showed that the DOX payload capability of MBs was determined by the proportion of DSPG. The DOX payload could affect the cavitation activities of MBs by reducing lipid fluidity, but this effect appeared to depend on the uniformity of the DOX distribution on the MB shell. Future studies will include combining this approach with acoustic radiation force or a disease-associated targeting ligand to locally concentrate MBs and further improve drug delivery and comparing the treatment outcomes with Lipodox in current clinical use.

## Figures and Tables

**Table 1:** Summary of MB formulations prepared to optimize DOX payload.

Group	DSPC:DSPG:DSPE-PEG2000 (Mole Ratio)
F1	84:0:2
F2	42:21:2
F3	42:42:2
F4	42:63:2
F5	42:84:2
F6	0:84:2

**Table 2:** Characteristics and operating frequency ranges of transducers.

Transducer	Central Frequency (MHz)	Focal Length (mm)	Bandwidth (−6 dB) (MHz)
V303-507938	1	18.0	0.6–1.2
V380-612225	3.5	51.6	1.6–4.6
V307-707444	5	49.6	2.9–7.6
V322-728004	10	52.1	6.0–12.4
V319-683709	15	50.5	10.1–18.6

## Data Availability

Not applicable.

## Supplementary Materials

The following are available online at https://www.mdpi.com/article/10.3390/pharmaceutics13122080/s1, Figure S1: The flowchart of calculating DOX distribution uniformity onto the MBs shell, Figure S2: The DOX retention time of different MBs formulations.
