# Supplementary material for: The Impact of Surface Drug Distribution on the Acoustic Behavior of DOX-Loaded Microbubbles
Source: Pharmaceutics. 2021 Dec 4;13(12):2080. doi: 10.3390/pharmaceutics13122080 (PMC8703561; doi:10.3390/pharmaceutics13122080)
Supplement: Supplementary file 1 [file pharmaceutics-13-02080-s001.zip › pharmaceutics-1469515-supplementary.pdf]

pharmaceutics

Supplementary Materials: The Impact of Surface Drug Distribution on the Acoustic Behavior of DOX-Loaded Microbubbles

Chia-Wei Lin , Ching-Hsiang Fan and Chih-Kuang Yeh *

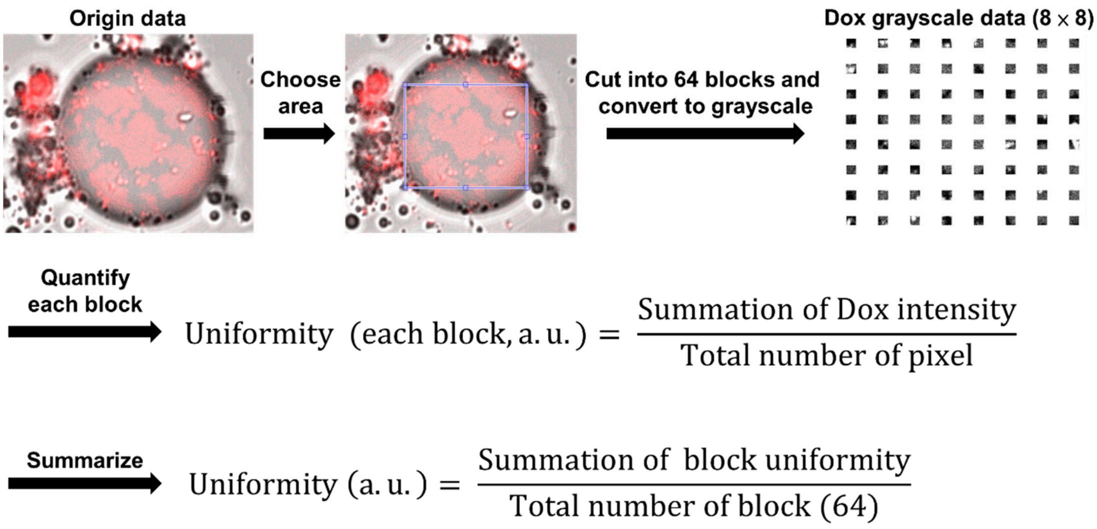

Figure S1. The flowchart of calculating DOX distribution uniformity onto the MBs shell.

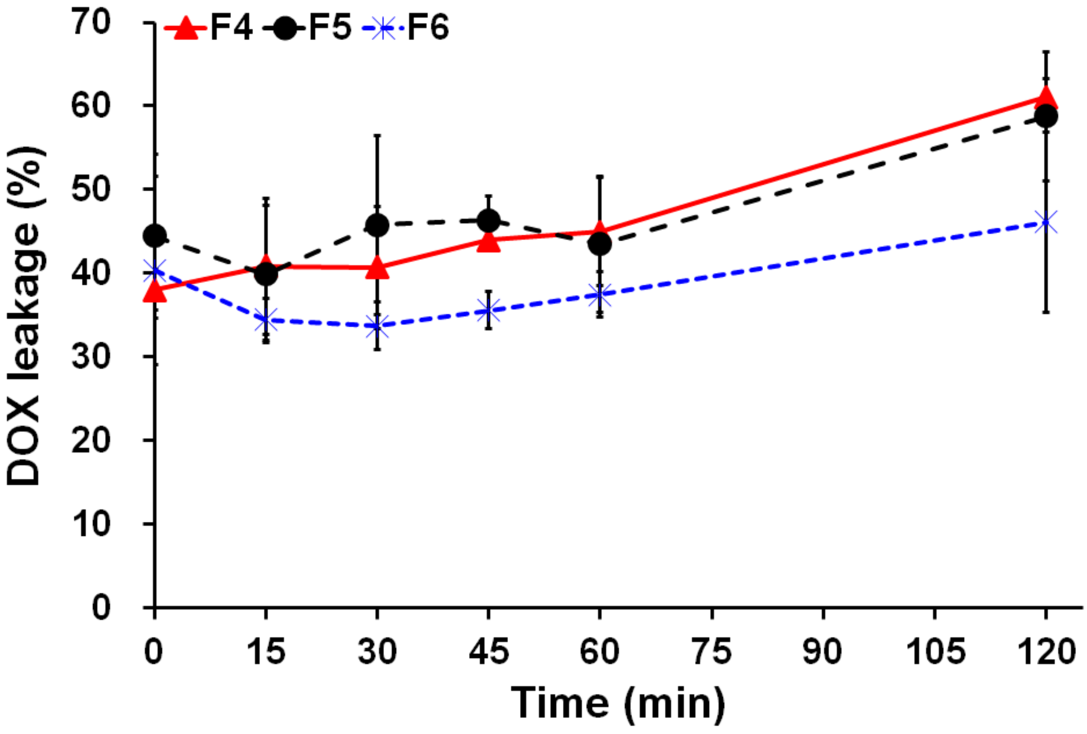

Figure S2. The DOX retention time of different MBs formulations.
